# Effect of Vitamin D_3_ Supplements on Development of Advanced Cancer: A Secondary Analysis of the VITAL Randomized Clinical Trial

**DOI:** 10.1001/jamanetworkopen.2020.25850

**Published:** 2020-11-18

**Authors:** Paulette D. Chandler, Wendy Y. Chen, Oluremi N. Ajala, Aditi Hazra, Nancy Cook, Vadim Bubes, I-Min Lee, Edward L. Giovannucci, Walter Willett, Julie E. Buring, JoAnn E. Manson

**Affiliations:** 1 Division of Preventive Medicine, Brigham and Women’s Hospital, Harvard Medical School, Boston, Massachusetts; 2 Department of Medical Oncology, Dana Farber Cancer Institute, Harvard Medical School, Boston, Massachusetts; 3 Channing Division of Network Medicine, Brigham and Women’s Hospital, Harvard Medical School, Boston, Massachusetts; 4 Department of Nutrition, Harvard T. H. Chan School of Public Health, Boston, Massachusetts; 5 Department of Epidemiology, Harvard T. H. Chan School of Public Health, Boston, Massachusetts

## Abstract

**Importance:**

Epidemiologic and trial data suggest that vitamin D supplementation may reduce metastatic cancer and cancer mortality, reflecting shared biological pathways.

**Objective:**

To follow up on the possible reduction in cancer death in the Vitamin D and Omega-3 Trial (VITAL) with an evaluation of whether vitamin D reduces the incidence of advanced (metastatic or fatal) cancer and an examination possible effect modification by body mass index.

**Design, Setting, and Participants:**

VITAL is a randomized, double-blind, placebo-controlled, 2 × 2 factorial clinical trial of vitamin D_3_ (cholecalciferol, 2000 IU/d) and marine omega-3 fatty acids (1 g/d). This multicenter clinical trial was conducted in the United States; participants included men aged 50 years or older and women aged 55 years or older who were free of cancer and cardiovascular disease at baseline. Randomization took place from November 2011 through March 2014, and study medication ended on December 31, 2017. Data for this secondary analysis were analyzed from November 1, 2011, to December 31, 2017.

**Interventions:**

Vitamin D_3_ (cholecalciferol, 2000 IU/d) and marine omega-3 fatty acids (1 g/d) supplements.

**Main Outcomes and Measures:**

For the present analysis, the primary outcome was a composite incidence of metastatic and fatal invasive total cancer, because the main VITAL study showed a possible reduction in fatal cancer with vitamin D supplementation and effect modification by body mass index (BMI; calculated as weight in kilograms divided by height in meters squared) for total cancer incidence reduction for individuals with normal BMI, but not for individuals with overweight or obesity. Secondary analyses included examination of BMI (<25, 25 to < 30, and ≥30) as effect modifiers of the observed associations.

**Results:**

Among 25 871 randomized VITAL participants (51% female; mean [SD] age, 67.1 [7.1] years), 1617 were diagnosed with invasive cancer over a median intervention period of 5.3 years (range, 3.8-6.1 years). As previously reported, no significant differences for cancer incidence by treatment arm were observed. However, a significant reduction in advanced cancers (metastatic or fatal) was found for those randomized to vitamin D compared with placebo (226 of 12 927 assigned to vitamin D [1.7%] and 274 of 12 944 assigned to placebo [2.1%]; HR, 0.83 [95% CI, 0.69-0.99]; *P* = .04). When stratified by BMI, there was a significant reduction for the vitamin D arm in incident metastatic or fatal cancer among those with normal BMI (BMI<25: HR, 0.62 [95% CI, 0.45-0.86]) but not among those with overweight or obesity (BMI 25-<30: HR, 0.89 [95% CI, 0.68-1.17]; BMI≥30: HR, 1.05 [95% CI, 0.74-1.49]) (*P* = .03 for interaction by BMI).

**Conclusions and Relevance:**

In this randomized clinical trial, supplementation with vitamin D reduced the incidence of advanced (metastatic or fatal) cancer in the overall cohort, with the strongest risk reduction seen in individuals with normal weight.

**Trial Registration:**

ClinicalTrials.gov Identifier: NCT01169259

## Key Points

**Question:**

Does vitamin D_3_ supplementation reduce the risk of developing advanced (metastatic or fatal) cancer among adults without a diagnosis of cancer at baseline?

**Findings:**

In this secondary analysis of a randomized clinical trial with 25 871 patients, supplementation with vitamin D_3_ reduced the incidence of advanced (metastatic or fatal) cancer in the overall cohort, with strongest risk reduction in individuals with normal weight and no reduction among individuals with overweight or obesity.

**Meaning:**

These findings suggest that vitamin D_3_ may reduce the risk of developing advanced cancer among adults without a diagnosis of cancer at baseline; this protective effect is apparent for those who have normal but not elevated body mass index.

## Teaser

This secondary analysis of a randomized clinical trial evaluates the effect vitamin D on the incidence of advanced (metastatic or fatal) cancer among adults without cancer at baseline and examines possible effect modification by body mass index.

## Introduction

Randomized trial data suggest a stronger benefit of vitamin D on cancer mortality and survival than cancer incidence.^1,2^ These data suggest that vitamin D may have a role in reducing more advanced or fatal cancers, but this specific question has not been previously addressed in randomized trials. Laboratory and animal studies show that vitamin D may inhibit carcinogenesis and slow tumor progression, including promotion of cell differentiation, inhibition of cancer cell proliferation, and anti-inflammatory, immunomodulatory, proapoptotic, and antiangiogenic effects.^1,2,3,4^ Vitamin D may decrease tumor invasiveness and propensity to metastasize, leading to reduced cancer mortality.^3^ Higher serum 25-hydroxyvitamin D (25[OH]D) levels at diagnosis have been linked to longer survival in cancer patients.^5^

Compared with placebo in the Vitamin D and Omega-3 Trial (VITAL),^6^ the hazard ratio (HR) for the vitamin D arm for incident total invasive cancer was 0.96 (95% CI, 0.88-1.06), but for total cancer mortality was 0.83 (0.67-1.02), suggesting a potential role of vitamin D in reducing metastatic or lethal cancers. Moreover, incident cancers were reduced in those with normal body mass index (BMI) but not in those with overweight or obesity, suggesting that factors associated with obesity may dampen the effect of vitamin D supplementation.^6^ Vitamin D supplementation may have different effects in patients with obesity vs without obesity on the basis of impaired immune function in obesity.^7,8^ Impaired immune function in the presence of obesity has been demonstrated in both humans and animal models.^9,10^ Obesity has been associated with chronic, low-grade inflammation and systemic dysregulation of natural killer cell (NK) function.^11,12^ Whether obesity is related to poorer tumor immunity is not well established, but some evidence suggests that immune checkpoint blockade therapy in cancer seems to work better in individuals with obesity.^13^ One theory is that individuals with obesity may have some defect, such as obesity-induced PD-1 expression and T cell exhaustion and dysfunction, that is corrected with immune checkpoint blockade.^14,15^ Thus, an intricate balance between adiposity and immunomodulatory or inflammatory mediators may contribute to the differential response to vitamin D_3_.^16^ We hypothesize that vitamin D_3_ supplementation reduces the incidence of metastatic cancer at diagnosis or lethal cancer and that the risk reduction is most pronounced in individuals with normal weight.

## Methods

### Study Design

**Figure 1.**
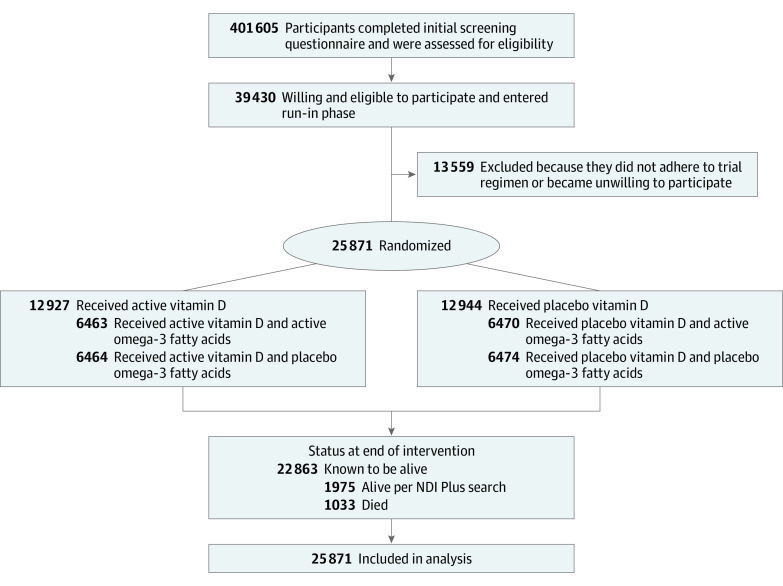
Screening, Randomization, and Follow-up of the Participants

The VITAL study was a randomized, double-blind, placebo-controlled, 2 × 2 factorial trial that examined the benefits and risks of vitamin D_3_ (cholecalciferol, 2000 IU/d) and marine omega-3 fatty acids (1 g/d) for primary prevention of cancer and cardiovascular disease among 25 871 participants (men aged ≥50 years and women aged ≥55 years). Figure 1 shows the 2-by-2 factorial trial design. Individuals were randomized to receive vitamin D_3_, marine omega-3 fatty acids, both active agents, or both placebos. The study protocol has been described in detail elsewhere.^11^ Supplement 1 contains (1) the original protocol, final protocol, and summary of changes and (2) the original statistical analysis plan, final statistical analysis plan, and summary of changes. This study followed the Consolidated Standards of Reporting Trials (CONSORT) reporting guideline for randomized clinical trials.^17^ The trial was approved by the institutional review board of Partners Healthcare/Brigham and Women’s Hospital and was monitored by an external Data and Safety Monitoring Board, and the study agents have received Investigational New Drug Approval from the US Food and Drug Administration. All participants provided written informed consent.

Participants were recruited throughout the US, balanced by sex, and with a goal to include at least 5000 Black participants. Eligible participants had no history of cancer (except nonmelanoma skin cancer) or cardiovascular disease at study entry. Other exclusion criteria included kidney failure or dialysis, cirrhosis, history of hypercalcemia, or other serious conditions that would preclude participation (Figure 1). Participants were required to agree to limit vitamin D to no greater than 800 IU/d from all supplemental sources, including multivitamins, and to forgo use of any out-of-study fish oil supplements. All final participants completed a 3-month placebo run-in phase (see Figure 1 for recruitment flow diagram). Randomization to vitamin D_3_, omega-3 fatty acids, both active agents, or both placebos took place from November 2011 to March 2014. Study medication ended as planned on December 31, 2017. The median intervention period was 5.3 years (range, 3.8-6.1 years). As previously reported, the mean rate of response to questionnaires was 93.1%, and follow-up regarding mortality was greater than 98% over the follow-up period.^6^ Nonstudy use of vitamin D (>800 IU/d) was low (3.8% and 6.4% in the vitamin D_3_ group and 5.6% and 10.8% in the placebo group at 2 years and 5 years, respectively).^6^ The mean rate of adherence (defined as taking at least two-thirds of trial capsules) was 82.0% in the vitamin D_3_ group and 80.3% in the placebo group.^6^

Baseline blood samples were collected during the run-in period from all willing participants, including 16 956 of 25 871 randomized (65.5%). Quest Diagnostics performed 25(OH)D assays on all analyzable samples using liquid chromatography–tandem mass spectrometry. Baseline 25(OH)D was used to evaluate effect modification by baseline 25(OH)D status on incident metastatic and fatal cancer. Participants received follow-up questionnaires at 6 months and 1 year after randomization and annually thereafter to collect information on adherence to randomized treatments, use of nonstudy vitamin D and fish oil supplements, development of major illnesses, cancer recurrence, updates on risk factors, and potential side effects of the study agents. Study capsules were mailed with questionnaires to participants. Baseline questionnaires collected data on risk factors for cancer, cardiovascular disease, and other conditions and included a food frequency questionnaire.

### Study End Points

The primary end points of this analysis were rates of the composite end point of metastatic and/or fatal cancer and time from baseline to metastatic/fatal cancer. As a secondary outcome, we examined effect modification by BMI based on vitamin D_3_ supplementation. This is an intention-to-treat analysis that includes participants lost to follow-up.

Participants reporting an end point were asked to sign a release for medical records, which were reviewed for confirmation by physicians blinded to treatment assignment. Cancer was confirmed with histologic or cytologic data.^18^ Participants were surveyed each year regarding cancer. Distant metastases were verified by medical record review at the time of diagnosis or when cause of death was verified as cancer. Analyses included only confirmed end points. For deaths reported by family members, the next-of-kin was asked for permission to obtain medical records and a copy of the death certificate. Alternatively, the latter was obtained from the state vital records bureau. Records were reviewed by physicians to assign cause of death. If records were unavailable (or participants lost to follow-up), the National Death Index Plus and Centers for Medicare and Medicaid Services databases were searched for cause of death based on death-certificate information.

### Statistical Analysis

Data were analyzed from November 1, 2011, to December 31, 2017. The trial sample size was determined to have greater than 85% power to detect hazard ratios of 0.85 and 0.80 for the primary end points of cancer and cardiovascular disease, respectively.

For this analysis, initial analyses compared baseline characteristics of participants according to randomized trial intervention with the use of *t* tests or χ^2^ tests. For the present analysis, the primary outcome was advanced cancer (a composite of metastatic and fatal invasive total cancer). We compared the main effects of vitamin D_3_ on metastatic and/or fatal cancer with the use of Cox proportional hazards models that were controlled for age, sex, and randomization group (omega-3 fatty acid group or placebo group). Person-time was counted from randomization to the end point, death, or the end of the trial on December 31, 2017. We included subjects lost to follow-up. Cumulative-incidence plots and interactions with time were used to examine whether effects varied over time. To assess for latent effects, we conducted sensitivity analyses by excluding the first 2 years of follow-up. A test for proportionality was performed to assess significance of difference in cumulative incidence curves.

Secondary analyses included examination of BMI (<25, 25-<30, and ≥30 kg/m^2^) as effect modifiers of the observed associations. We assessed overall and site-specific differences in metastatic and fatal cancer and time from baseline to cancer death based on vitamin D_3_ supplementation and BMI. We assessed whether BMI was still balanced by randomization group after stratifying by median 25(OH)D (<31 ng/mL vs ≥31 ng/mL). We used the *t* test to compare the mean BMI for active vitamin D vs placebo within the baseline subgroups of above and below median 25(OH)D. A 2-sided *P* < .05 was considered statistically significant.

Additional secondary outcomes included the influence of vitamin D_3_ supplementation on site-specific (breast, prostate, colorectal, and lung) metastatic and fatal cancer and variations in the effect according to race or ethnic group and baseline vitamin D status. However, there was no control for multiple hypothesis testing, and no formal adjustment was made to the *P* values or confidence intervals. Thus, results regarding secondary end points should be interpreted with caution.

## Results

**Figure 2.**
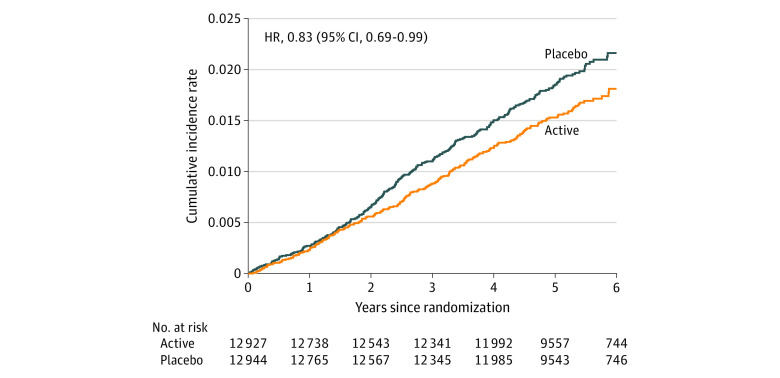
Vitamin D (Active) and Placebo: Cumulative Incidence Rates of Metastatic and Fatal Cancer of Any Type HR indicates hazard ratio.

This study included 25 871 randomized VITAL participants (51% women) with mean (SD) age of 67.1 (7.1) years. There were no significant differences in the baseline characteristics between the vitamin D_3_ and placebo groups (Table 1). Participants were balanced by sex, were racially/ethnically diverse (including 20.2% African American/Black), and had a mean (SD) BMI of 28.1 (5.7) kg/m^2^. Overall, 11 030 individuals (43%) across both the vitamin D_3_ and placebo groups were taking supplemental vitamin D (allowed up to the recommended dietary allowance, ≤800 IU/d). (Table 1) Overall, participants were not vitamin D deficient (mean 25(OH)D − 30 ng/ml in both vitamin D and placebo groups). (Table 1)^12^ There were no significant differences between the vitamin D and placebo groups with respect to incident diagnoses of hypercalcemia, kidney stones, or gastrointestinal symptoms.^6^ Of the 25 871 VITAL participants, 1617 were diagnosed with invasive cancer over a median 5.3-year intervention period (range, 3.8-6.1 years). As previously reported, no significant differences by treatment arm were observed for incident cancer (vitamin D_3_ vs placebo: hazard ratio [HR] = 0.96; 95% CI, 0.88-1.06; *P* = .47) or cancer mortality (HR = 0.83; 95% CI, 0.67-1.02]; *P* = .08).^6^ Of 17 site-specific cancers examined separately, only uterine cancer (in 35 patients assigned to vitamin D_3_ [0.3%] and 20 assigned to placebo [0.2%]; HR, 1.75; 95% CI, 1.01-3.03; *P* = .046) showed significant differences by treatment group (eTable 1 in Supplement 2). However, a significant reduction in advanced cancers (metastatic or fatal) was found for those randomized to vitamin D compared with placebo (in 226 of 12 927 assigned to vitamin D_3_ [1.7%] and in 274 of 12 944 assigned to placebo [2.1%]; HR, 0.83; 95% CI, 0.69-0.99; *P* = .04) (Table 2). The cumulative incidence rate of total metastatic and fatal cancer is shown in Figure 2. The vitamin D_3_ vs placebo curves start to diverge at 2 years; the test for proportionality over time was not significant Results were similar even after excluding the first 2 years of follow-up. (Table 2) Among the 12 927 participants assigned to vitamin D treatment, 16 had incident metastatic cancer and also died from cancer during the trial period; among the 12 944 participants randomized to vitamin D placebo, 24 with incident metastatic cancer died from cancer within the randomization period. There was no association of omega-3 fatty acid supplementation with advanced cancer, nor was there an interaction by omega-3 treatment arm.

**Table 1.** Baseline Characteristics

Baseline Characteristic	No. (%)^a^		
	All participants (N = 25 871)	Vitamin D	
		Active (n = 12 927)	Placebo (n = 12 944)
Female	13 085 (51)	6547 (51)	6538 (51)
Age, mean (SD), y	67.1 (7.1)	67.1 (7.0)	67.1 (7.1)
Race/ethnicity^b^			
Non-Hispanic White	18 046 (71)	9013 (71)	9033 (71)
Black	5106 (20)	2553 (20)	2553 (20)
Nonblack Hispanic	1013 (4)	516 (4)	497 (4)
Asian or Pacific Islander	388 (2)	188 (2)	200 (2)
American Indian or Alaska Native	228 (1)	118 (1)	110 (1)
Other or unknown	523 (2)	259 (2)	264 (2)
Body mass index, mean (SD)^c^	28.1 (5.7)	28.1 (5.7)	28.1 (5.8)
<25	7843 (31)	3884 (31)	3959 (31)
25-<30	10 122 (40)	5060 (40)	5062 (40)
≥30	7289 (29)	3679 (29)	3610 (29)
Current smoking	1836 (7)	921 (7)	915 (7)
Diabetes	3549 (14)	1812 (14)	1737 (13)
Any alcohol use^d^	17 443/25 437 (69)	8726/12 703 (69)	8717/12 734 (68)
Current use of supplemental vitamin D, ≤800 IU/d^e^	11 030 (43)	5497 (43)	5533 (43)
Baseline 25(OH)D, mean (SD), ng/mL^f^	30.8 (10.0)	30.9 (10.0)	30.8 (10.0)
Cancer screening at baseline			
Mammography or breast biopsy within past 10 y among women only	12 212 (94)	6089 (94)	6123 (94)
Colonoscopy or other colon cancer screening within past 10 y	23 055 (90)	11 523 (90)	11 532 (90)
PSA screening within past 10 y, among men only	9586 (77)	4771 (77)	4815 (77)

SI conversion factor: To convert 25(OH)D from ng/mL to nmol/L, multiply by 2.5.

^a^ Percentages may not sum to 100 because of rounding or because of missing values for cancer screening. There were no significant differences between the groups with regard to the baseline characteristics.

^b^ Race and ethnicity were reported by the participants.

^c^ Calculated as weight in kilograms divided by height in meters squared. Data were missing for 2.4% of the participants.

^d^ Includes any alcohol use, at least monthly.

^e^ From all supplemental sources of vitamin D combined (individual vitamin D supplements, calcium + vitamin D supplements, medications with vitamin D, and multivitamins).

^f^ N = 15 787 with measured values.

**Table 2.** Hazard Ratios and 95% CIs of Total and Site-Specific Cancers and Mortality by Randomized Vitamin D^a^

Disease outcome	No. of events		HR (95% CI)	*P* value
	Vitamin D (N = 12 927)	Placebo (N = 12 944)		
Confirmed cancer				
Total invasive	793	824	0.96 (0.88-1.06)	.47
Breast	124	122	1.02 (0.79-1.31)	.90
Prostate	192	219	0.88 (0.72-1.07)	.19
Colorectal	51	47	1.09 (0.73-1.62)	.67
Lung	74	74	1.00 (0.73-1.38)	.99
Confirmed metastatic cancer of any type	88	111	0.80 (0.60-1.05)	.11
Total cancer mortality	154	187	0.83 (0.67-1.02)	.08
Confirmed metastatic cancer or cancer death of any type	226	274	0.83 (0.69-0.99)	.04
Excluding 1 y	193	235	0.82 (0.68-1.00)	.047
Excluding 2 y	147	181	0.81 (0.66-1.01)	.07

Abbreviation: HR, hazard ratio.

^a^ Analyses were from Cox regression models that were controlled for age, sex, and omega-3 fatty acid randomization group. Analyses were not adjusted for multiple comparisons. Analyses were done as intention-to-treat over all years of follow-up.

Because prostate cancer was associated with a lower but nonsignificant reduction in incidence with vitamin D supplementation (HR, 0.88; 95% CI, 0.72-1.07; *P* = .19) and was the largest contributor to total cancer incidence, we conducted sensitivity analyses excluding metastatic /fatal prostate cancer. Results were similar (HR, 0.85; 95% CI, 0.71-1.02; *P* = .08). Case numbers for the site-specific HRs for breast, prostate, colorectal, and lung mortality are too small to be interpreted (eTable 2 in Supplement 2).

When stratified by BMI, there was a significant reduction for the vitamin D_3_ arm in incident metastatic or fatal cancer among those with normal BMI (BMI <25: HR, 0.62; 95% CI, 0.45-0.86), but not among those with overweight or obesity (BMI 25 to <30: HR, 0.89; 95% CI, 0.68-1.17; BMI ≥30: HR, 1.05; 95% CI, 0.74-1.49; *P* = .03 for interaction) (Table 3). There was a stepwise decrease in effect size of the association of vitamin D treatment with metastatic cancer and cancer mortality by each higher BMI subgroup. The effect sizes for BMI<25 were similar for total cancer death, metastatic cancer, and the composite end point of advanced cancer(Table 3). Whereas mean BMI varied by baseline 25(OH)D (25[OH]D <31 ng/mL: mean [SD] BMI in the vitamin D group, 29.0 [6.0]; in the placebo group, 28.9 [6.0] [*P* = .46]; 25[OH]D ≥31 ng/mL: mean [SD] BMI in the vitamin D group, 26.9 [5.1]; in the placebo group, 26.7 [5.0] [*P* = .05]), BMI remained balanced by treatment group after stratifying by 25(OH)D.

**Table 3.** Vitamin D, Total Metastatic and Cancer Mortality Hazard Ratios and 95% Confidence Intervals by BMI Categories^a^

BMI category	Total No.	No. events in groups		Hazard ratio		*P* value for interaction
		Vitamin D	Placebo	HR (95% CI)	*P* value	
**Total metastatic cancer and cancer mortality (n = 25 254)**						
<25	7843	58	96	0.62 (0.45-0.86)	.004	.03
25 to <30	10 122	98	109	0.89 (0.68-1.17)	.42	
≥30	7289	65	61	1.05 (0.74-1.49)	.79	
**Total metastatic cancer (n = 25 254)**						
<25	7843	24	39	0.63 (0.38-1.05)	.08	.21
25 to <30	10 122	37	46	0.80 (0.52-1.23)	.31	
≥30	7289	25	24	1.03 (0.59-1.80)	.92	
**Cancer mortality (n = 25 254)**						
<25	7843	38	68	0.58 (0.39-0.86)	.007	.02
25 to <30	10 122	66	74	0.89 (0.64-1.23)	.472	
≥30	7289	46	39	1.15 (0.75-1.76)	.518	

Abbreviations: BMI, body mass index; HR, hazard ratio.

^a^ Analyses were from Cox regression models that were controlled for age, sex, and omega-3 fatty acid randomization group. Analyses were not adjusted for multiple comparisons.

In analyses stratified by race, no effect modification was observed. Non-Hispanic White participants (163 assigned to vitamin D_3_ and 205 assigned to placebo; HR, 0.80; 95% CI, 0.65-0.98; *P* = .03) and Black participants (40 assigned to vitamin D_3_ and 46 assigned to placebo; HR, 0.86; 95% CI, 0.56-1.32; *P* = .49) had a similar risk reduction for total metastatic cancer/cancer mortality (eTable 3 in Supplement 2).

For individuals with low serum 25(OH)D levels (<20 ng/mL) (n = 2001), the rate of metastatic/fatal cancer outcomes (in 20 participants assigned to vitamin D and 25 assigned to placebo; HR, 0.85; 95% CI, 0.47-1.54) was similar to the rate among individuals with serum 25(OH)D levels greater than or equal to 20 ng/mL (n = 13 786) (in 124 participants assigned to vitamin D and 140 assigned to placebo; HR 0.88; 95% CI, 0.69-1.12) (*P* = .95 for interaction).

Similarly, no significant interaction by baseline 25(OH)D level was observed for individuals with 25(OH)D levels below median 25(OH)D (<31 ng/mL) (n = 7812) for metastatic/fatal cancer outcomes (in 79 participants assigned to vitamin D and 77 assigned to placebo; HR 1.05; 95% CI, 0.77-1.44) vs individuals with serum 25(OH)D greater than or equal to 31 ng/mL (n = 7975) (in 65 participants assigned to vitamin D and 88 assigned to placebo; HR 0.72; 95% CI, 0.52-1.00) (*P* = .10 for interaction).

## Discussion

In this more detailed secondary analysis of VITAL, vitamin D_3_ reduced the risk of developing advanced (metastatic or fatal) cancer among adults without a diagnosis of cancer at baseline. However, this protective effect was apparent only for those with normal BMI. We did not see differences in effect by race or baseline vitamin D levels. Our findings are not due to one particular cancer, because a broad mix of cancers contributed. Removing prostate cancer from analyses did not attenuate the observed effect of vitamin D supplementation on advanced cancer, suggesting the results were not driven by prostate cancer alone. Our findings suggest that vitamin D supplementation may be operating through a general, rather than site-specific, mechanism to reduce the risk of advanced cancer.

The first 2 randomized clinical trials of vitamin D_3_ supplementation in cancer patients were mixed. The SUNSHINE trial^19^ compared the addition of high-dose vitamin D_3_ (vitamin D_3_ 8000 IU/d for 2 weeks and 4000 IU/d thereafter) vs standard-dose vitamin D_3_ (400 IU/d) in conjunction with standard chemotherapy in 139 patients with advanced or metastatic colorectal cancer (median follow-up of 22.9 months) with a nonsignificant improvement in progression-free survival (13.0 vs 11.0 months; *P* = .07) but a decreased risk of progression-free survival or death (HR, 0.74; *P* = .02). Furthermore, the effect of high-dose vitamin D_3_ on improvement in progression-free survival seemed to be greater among patients with a lower BMI (*P* = .04 for interaction). However, this study was small and underpowered. The AMATERASU trial^20^ included 417 patients with stage I to III digestive tract cancer randomized to vitamin D_3_ (2000 IU/d) or placebo. Vitamin D supplementation resulted in no significant improvement in relapse-free survival at 5 years. However, the AMATERASU study had a treatment allocation imbalance for age, and post hoc age-adjusted analysis revealed a statistically significant benefit in favor of supplementation (relapse-free survival HR, 0.66; 95% CI, 0.43-0.99).^20^ A recent meta-analysis of randomized clinical trials compared people who took vitamin D supplements with those who took a placebo for at least 3 years; people who took vitamin D supplements had a 13% lower risk of dying from cancer than those who took a placebo (*P* = .005), which was largely attributable to interventions with daily dosing (as opposed to bolus dosing).^1^ Another meta-analysis of vitamin D clinical trials showed that vitamin D supplementation reduced the risk of cancer death by 16%, and all-cause mortality was significantly lower in trials with vitamin D_3_ supplementation vs vitamin D2 supplementation.^2^ Our findings along with previous randomized trials support the ongoing evaluation of vitamin D supplementation for metastatic cancer. An association between vitamin D supplementation and metastatic and fatal cancer is biologically plausible. Vitamin D receptors are widely expressed throughout the body, and experimental evidence suggests that vitamin D has antineoplastic activity.^21^ The binding of vitamin D to the vitamin D receptor results in transcriptional activation and repression of target genes, producing apoptosis,^21^ antiproliferative effects,^22^ and immunomodulatory effects that may contribute to reduced metastatic disease^21^ and fatal cancer.^23^ A meta-analysis of prospective cohort studies showed that higher 25(OH)D concentration was associated with 19% lower risk of cancer mortality, and the risk of cancer mortality was 2% lower with each 20 nmol/L increment of 25(OH)D concentration.^24^ Vitamin D deficiency prevalence is high in cancer patients,^25,26^ with 1 study reporting vitamin D deficiency in 72% of cancer patients.^19,27^ Known risk factors for vitamin D deficiency are common in in this population, including female sex, low sunlight exposure, being under palliative care, receiving adjuvant chemotherapy, or history of gastrointestinal surgery.^27^

Interestingly, we found significant effect modification by BMI. The effect modification was not due to greater power to detect a protective effect in the healthy BMI group. The healthy BMI group had the lowest absolute rate of cancer (only 31% of the incident cancers were in the participants with BMI<25). The statistical power was actually higher among those with elevated BMIs.

A dynamic interplay between adiposity and immunomodulatory or inflammatory mediators may contribute to the differential response to vitamin D_3_. In the initial VITAL publication, 2000 IU/d of vitamin D was associated with lower total cancer incidence among participants with normal BMI (HR, 0.76; 95% CI, 0.63-0.90) but not among those with overweight (HR, 1.04; 95% CI, 0.90-1.21), or obesity (HR, 1.13; 95% CI, 0.94-1.37).^6^ Similarly, we found that baseline serum 25(OH)D levels did not modify the effect of vitamin D supplementation on incident metastatic/fatal cancer. These results are congruent with the initial VITAL analysis, in which baseline 25(OH)D was not an effect modifier for incident total invasive cancer (25[OH]D < median of 31 ng/mL vs 25[OH]D ≥ median of 31 ng/mL, *P* = .57 for interaction) whereas BMI was a significant effect modifier for total cancer (*P* = .002 for interaction). Furthermore, higher serum 25(OH)D was correlated with lower BMI.

Although these findings could be due to chance, obesity is known to affect the vitamin D axis.^28^ The larger storage capacity for vitamin D in individuals with obesity by fat sequestration^29^ or volumetric dilution^30^ may result in lower plasma vitamin D. Yet in the overall VITAL cohort, neither individuals with or without obesity were deficient in vitamin D following vitamin D supplementation.^6^ Parathyroid hormone level, a marker of vitamin D efficacy, is higher in individuals with obesity compared with lean individuals at a given 25(OH)D level,^31,32^ which would be consistent with obesity-related hormonal dysregulation and less supplementation benefit.^31^ Low levels of 25-hydroxyvitamin D_3_ (<20 ng/mL) trigger a compensatory increase in parathyroid hormone levels, which accelerates bone resorption and stabilizes calcium. When vitamin D levels are deficient, vitamin D supplementation usually leads to reduction in serum parathyroid hormone levels in individuals with normal weight. However, the dose of vitamin D supplementation for the suppression of parathyroid hormone levels may differ in adults with overweight and obesity.^33^

Alternatively, because of volumetric dilution^30^ or decreased bioactivity of vitamin D, persons with overweight or obesity may require higher doses to derive cancer benefit, analogous to body size differences in aspirin dosage requirements.^34^ Moreover, prior research points to other mechanisms through which vitamin D supplementation might reduce cancer risk in participants with normal weight but not those with overweight or obesity. Vitamin D may modulate NK activity; dietary vitamin D supplementation increased NK activity in lean, but not in obese mice.^12^ Similarly, a study reported impaired NK cell phenotype and NK cell subset alterations in obese individuals vs lean individuals.^11^

### Strengths and Limitations

Our study has several strengths, including a large general population sample with racial/ethnic and geographic diversity, daily vitamin D dosing, high follow-up rates and pill-taking adherence, rigorously adjudicated end points, baseline and follow-up blood collections in many participants, and achieved mean 25(OH)D levels in the targeted range. Limitations of our study warrant consideration, however. Power is still limited at this point, but these preliminary data may inform current ongoing studies with vitamin D. As a primary prevention trial, VITAL studied 2000 IU per day, but higher doses could be considered for future studies. Median treatment duration was 5.3 years, but this is a common duration for adjuvant treatments used in cancers, including breast cancer. Ongoing trials^35,36^ will add information regarding other doses, although some are using bolus dosing. A 2-year postintervention follow-up of our cohort is ongoing to capture later events and increase statistical power to assess end points.

## Conclusions

In summary, this randomized clinical trial of daily high-dose vitamin D supplementation for 5 years reduced the incidence of advanced (metastatic or fatal) cancer in the overall cohort of adults without a diagnosis of cancer at baseline, with strongest risk reduction in individuals with normal weight. Additional randomized trials focusing on cancer patients should be considered, as well as investigations of differential benefit by BMI. Even if vitamin D effects were modest, vitamin D supplementation at the studied levels are much less toxic and lower cost than many current cancer therapies.

## Notes

Supplement 1. **Trial Protocol**

Supplement 2. **eTable 1.** Hazard Ratios (HR) and 95% Confidence Intervals (CI) of Other Cancers by Randomized Vitamin D, All Years of Follow-up **eTable 2.** Hazard Ratios (HR) and 95% Confidence Intervals (CI) of Specific Cancers and Mortality by Randomized Vitamin D **eTable 3.** Randomized Vitamin D, Hazard Ratios (HR) and 95% Confidence Intervals (CI) for Total Metastatic and Cancer Mortality by Race

Supplement 3. **Data Sharing Statement**
