# Error in Conflict of Interest Disclosures

**DOI:** 10.1001/jamanetworkopen.2020.32460

**Published:** 2020-12-11

In the Original Investigation titled “Effect of Vitamin D_3_ Supplements on Development of Advanced Cancer: A Secondary Analysis of the VITAL Randomized Clinical Trial,”^1^ published online November 18, 2020, the Conflict of Interest Disclosures section was missing grants for author Oluremi Ajala from the National Heart, Lung, and Blood Institute of the National Institutes of Health. This article has been corrected.
