# Supplementary material for: Effect of Vitamin D3 Supplements on Development of Advanced Cancer: A Secondary Analysis of the VITAL Randomized Clinical Trial
Source: JAMA Netw Open. 2020 Nov 18;3(11):e2025850. doi: 10.1001/jamanetworkopen.2020.25850 (PMC7675103; doi:10.1001/jamanetworkopen.2020.25850)
Supplement: Supplement 2. — eTable 1. Hazard Ratios (HR) and 95% Confidence Intervals (CI) of Other Cancers by Randomized Vitamin D, All Years of Follow-up eTable 2. Hazard Ratios (HR) and 95% Confidence Intervals (CI) of Specific Cancers and Mortality by Randomized Vitamin D eTable 3. Randomized Vitamin D, Hazard Ratios (HR) and 95% Confidence Intervals (CI) for Total Metastatic and Cancer Mortality by Race [file jamanetwopen-e2025850-s002.pdf]

Supplemental Online Content

Chandler PD, Chen WY, Ajala ON, et al; VITAL Research Group. Effect of vitamin D_3 supplements on development of advanced cancer: a secondary analysis of the VITAL randomized clinical trial. *JAMA Netw Open*. 2020;3(11):e2025850. doi:10.1001/jamanetworkopen.2020.25850

eTable 1. Hazard Ratios (HR) and 95% Confidence Intervals (CI) of Other Cancers by Randomized Vitamin D, All Years of Follow-up

eTable 2. Hazard Ratios (HR) and 95% Confidence Intervals (CI) of Specific Cancers and Mortality by Randomized Vitamin D

eTable 3. Randomized Vitamin D, Hazard Ratios (HR) and 95% Confidence Intervals (CI) for Total Metastatic and Cancer Mortality by Race

This supplemental material has been provided by the authors to give readers additional information about their work.

© 2020 Chandler PD et al. *JAMA Network Open.*

eTable 1. Hazard Ratios (HR) and 95% Confidence Intervals (CI) of Other Cancers by Randomized Vitamin D, All Years of Follow-up[a]

	No of Events				
**Outcome**	**Active Treatment (N=12,927)**	**Placebo (N=12,944)**	**HR**	**95%CI**	**P-value**
**Confirmed Cancer + Cancer Death**					
Bladder Cancer	13	23	0.57	0.29-1.12	0.10
Brain Cancer	10	11	0.91	0.39-2.15	0.83
Cervical Cancer	2	3	0.67	0.11-4.01	0.66
Esophageal Cancer	10	12	0.84	0.36-1.95	0.69
Other female genital	2	4	0.50	0.09-2.73	0.43
Pancreatic Cancer	28	32	0.88	0.53-1.46	0.61
Stomach Cancer	13	7	1.86	0.74-4.67	0.18
Thyroid Cancer	10	12	0.83	0.36-1.93	0.67
Uterine Cancer	35	20	1.75	1.01-3.03	0.046
Kidney Cancer	18	23	0.78	0.42-1.45	0.44
Leukemia Cancer	22	22	1.00	0.56-1.81	0.99
Liver Cancer	7	12	0.59	0.23-1.49	0.26
Lung Cancer	74	74	1.00	0.73-1.38	0.99
Lymphoma Cancer	23	32	0.72	0.42-1.23	0.24
Melanoma Cancer	51	53	0.96	0.66-1.42	0.85
Multiple Myeloma Cancer	10	15	0.67	0.30-1.49	0.33
Ovarian Cancer	8	9	0.89	0.34-2.31	0.81

[a]Analyses were from Cox regression models that were controlled for age, sex, and omega-3 fatty acid randomization group. Analyses were not adjusted for multiple comparisons. Analyses were done as intention-to-treat over all years of follow-up

© 2020 Chandler PD et al. *JAMA Network Open.*

eTable 2. Hazard Ratios (HR) and 95% Confidence Intervals (CI) of Specific Cancers and Mortality by Randomized Vitamin D[a]					
	No of Events				
Disease Outcome	Vitamin D (N=12,927)	Placebo (N=12,944)	HR	95% CI	P-value
**Confirmed Metastatic Cancer**					
Metastatic breast cancer	2	2	1.00	0.14–7.09	1.00
Metastatic prostate cancer	3	8	0.38	0.10–1.42	0.15
Metastatic colorectal cancer	6	4	1.51	0.42–5.34	0.53
**Death**					
Death from breast cancer	6	3	2.00	0.50–7.99	0.33
Death from prostate cancer	3	9	0.34	0.09–1.24	0.10
Death from colorectal cancer	9	14	0.65	0.28–1.50	0.31
**Confirmed Metastatic Cancer or Cancer**					
Metastatic breast cancer / breast cancer death	6	4	1.50	0.42–5.30	0.53
Metastatic prostate cancer / prostate cancer death	6	14	0.43	0.17–1.12	0.09
Metastatic colorectal cancer / colorectal cancer death	12	15	0.81	0.38–1.73	0.58

[a]Analyses were from Cox regression models that were controlled for age, sex, and omega-3 fatty acid randomization group. Analyses were not adjusted for multiple comparisons. Analyses were done as intention-to-treat over all years of follow-up

© 2020 Chandler PD et al. *JAMA Network Open.*

eTable 3. Randomized Vitamin D, Hazard Ratios (HR) and 95% Confidence Intervals (CI) for Total Metastatic and Cancer Mortality by Race[a]

		No. Events in Groups		Hazard Ratio		P-Interaction
**Subgroup Category**	**Total**	**Vitamin D**	**Placebo**	**HR (95%CI)**	**P-value**	**P-value**
	**Total Metastatic Cancer/ Cancer Mortality**					
**Race**	**25,304**					0.94
Non-Hispanic White	18,046	163	205	0.80 (0.65–0.98)	0.03	
Black	5,106	40	46	0.86 (0.56–1.32)	0.49	
Other	2,152	16	19	0.86 (0.44-1.66)	0.65	
	**Total Metastatic Cancer**					
**Race**	**25,304**					0.25
Non-Hispanic White	18,046	69	91	0.76 (0.56–1.04)	0.09	
Black	5,106	6	13	0.46 (0.17–1.20)	0.11	
Other	2,152	9	6	1.50 (0.53-4.22)	0.44	
	**Cancer Mortality**					
**Race**	**25,304**					0.40
Non-Hispanic White	18,046	106	135	0.79 (0.61–1.02)	0.07	
Black	5,106	37	35	1.06 (0.67–1.68)	0.81	
Other	2,152	8	14	0.58 (0.24-1.38)	0.22	

[a]Analyses were from Cox regression models that were controlled for age, sex, and omega-3 fatty acid randomization group. Analyses were not adjusted for multiple comparisons. Analyses were done as intention-to-treat over all years of follow-up.

© 2020 Chandler PD et al. *JAMA Network Open.*
