# Supplementary material for: Effect of Vitamin D3 Supplements on Development of Advanced Cancer: A Secondary Analysis of the VITAL Randomized Clinical Trial
Source: JAMA Netw Open. 2020 Nov 18;3(11):e2025850. doi: 10.1001/jamanetworkopen.2020.25850 (PMC7675103; doi:10.1001/jamanetworkopen.2020.25850)
Supplement: Supplement 3. — Data Sharing Statement [file jamanetwopen-e2025850-s003.pdf]

Data Sharing Statement

Chandler. Effect of Vitamin D_3 Supplements on Development of Advanced Cancer. *JAMA Netw Open*. Published November 18, 2020.
10.1001/jamanetworkopen.2020.25850

Data
Data available: Yes
Data types: Deidentified participant data
How to access data: request for data must be sent to an individual.JoAnn E. Manson; jmanson@partners.org
When available: beginning date: 12-01-2021

Supporting Documents
Document types: None

Additional Information
Who can access the data: researchers whose proposed use of the data has been approved
Types of analyses: for a specified purpose
Mechanisms of data availability: with a signed data access agreement
Any additional restrictions: n/a
